# Not all mindfulness is equal: certain facets of mindfulness have important implications for well-being and mental health across the lifespan

**DOI:** 10.3389/fpsyg.2024.1347487

**Published:** 2024-04-15

**Authors:** Nathaniel J. Johnson, Ryan J. Smith, Hali Kil

**Affiliations:** Department of Psychology, Simon Fraser University, Burnaby, BC, Canada

**Keywords:** mindfulness, well-being, mental health, lifespan, latent profile analysis

## Abstract

The connections between the five facets of mindfulness, well-being, and mental health across the lifespan have traditionally been investigated using variable-centered approaches. Less research has investigated these relationships from a person-centered, profile-based approach. In this work, we aimed to identify the profiles of mindfulness in a Canadian lifespan sample (14 to 90 years of age) and investigate how these profiles compared on age, well-being, and mental health. An age- and gender-balanced sample of 1,600 participants completed a questionnaire that measured the five facets of mindfulness; life satisfaction; existential well-being; and anxiety, depression, and stress symptoms. A latent profile analysis was conducted. Five profiles based on the five-facet model of mindfulness were identified: *high mindfulness, moderate mindfulness, low mindfulness, nonjudgmentally aware,* and *judgmentally observing*. The 3-step approach to profile comparisons was used to assess age, mental health, and well-being differences across the profiles. Those in the high mindfulness and nonjudgmentally aware profiles were generally older, while the judgmentally observing profile contained younger individuals. Those in the high mindfulness and nonjudgmentally aware profiles reported the greatest mental health and well-being. Conversely, those in the low mindfulness and judgmentally observing profiles had worse mental health than the other profiles. The moderate mindfulness profile was situated between these profile groups on age, mental health, and well-being outcomes. This pattern of results has implications for mindfulness-based intervention research and practice to better account for heterogeneity in mindfulness and better support well-being across the lifespan.

## Introduction

The concept of mindfulness originally derived from Buddhist philosophy as a state of being characterized by attention to the present moment in a way that consciously promotes ethical action and non-attachment (Gethin, 2015). In Buddhist contexts, mindfulness was thought to lead to the enlightenment of the sincere follower (Gethin, 2015). Today, in contemporary Western psychology, mindfulness is considered a secular construct that can be embraced by individuals with a wide range of backgrounds. To Western psychologists, mindfulness has often been defined as the attention to and awareness of present-moment experiences, emotions, sensations, and perceptions with a non-reactive and non-judgmental attitude (Kabat-Zinn, 2003; Bishop et al., 2004).

In this secular context, some scholars consider mindfulness to be multidimensional; one of the most recognized operationalizations characterizes mindfulness as incorporating five facets (Baer et al., 2006, 2008). These facets are *observing,* the awareness of and attention to internal and external experiences such as emotions, sensations, and perceptions; *describing,* the ability to label experiences with language; *acting with awareness*, behaving in ways that are attentive to the present moment; *non-judging of inner experiences,* an attitude characterized by non-evaluation of inner thoughts and emotions; and *non-reactivity to inner experiences,* the ability to allow inner thoughts and emotions to come and go without getting caught up in them. Based on the five-facet model, Baer et al. (2008) established the Five Facet Mindfulness Questionnaire (FFMQ) as a self-report measure of the facets.

Mindfulness, as captured by the FFMQ, has been found to connect to various well-being outcomes such as decreased anxiety and depressive symptomology (Tomlinson et al., 2018), increased satisfaction with life (Mattes, 2019), and increased happiness (Campos et al., 2016). Furthermore, the well-being benefits of cultivating mindfulness have been supported across the lifespan from youth and adolescents (e.g., Zoogman et al., 2015; Reangsing et al., 2021), to older adult populations (e.g., Geiger et al., 2016).

Despite these findings, research suggests that not all mindfulness facets behave uniformly. For example, a recent meta-analysis found that all facets of mindfulness except observing negatively correlated with maladaptive affective symptomology (Carpenter et al., 2019). Furthermore, some facets relate to well-being in different ways across different samples of interest (Bravo et al., 2018). In Baer et al.’ (2008) original validation of the FFMQ, the observing facet positively correlated with psychological symptoms in college students without meditation experience and negatively correlated with the same symptoms in those with meditation experience. These inconsistencies have also been found across age groups. For example, Royuela-Colomer and Calvete (2016) found no significant correlation between the non-reactivity facet and depressive symptoms in a sample of adolescents, whereas Jones et al. (2022) found a significant negative correlation between non-reactivity and depressive symptoms in young adults. The variability in findings across mindfulness facets makes it difficult to develop a clear understanding of how mindfulness connects to well-being. Therefore, a deeper examination of facet-level heterogeneity among lifespan samples is necessary to more fully understand how individual-level variability in mindfulness relates to adaptive outcomes.

The majority of studies that have investigated mindfulness and well-being have taken variable-centered approaches that assume a sample of interest is derived from a single population. Variable-centered analyses (e.g., regression, ANOVA) are largely based on mean scores that are generalized across participants to infer something about a population. Given the heterogeneous results based on mindfulness facets and across different samples in the literature, some scholars have argued that the construct should also be explored using person-centered approaches (e.g., Lilja et al., 2013; Pearson et al., 2015; Bravo et al., 2016; Lecuona et al., 2022). Person-centered analyses (e.g., cluster analyses, latent profile analyses) aim to identify subgroups within a given sample. Applying these approaches to FFMQ research yields different groups (i.e., clusters, profiles) based on heterogeneous score combinations across the five mindfulness facets for individual respondents. Person-centered approaches could address some of the inconsistencies in previous findings as certain combinations of mindfulness facets may be more adaptive than others.

Since Lilja et al.’s (2013) and Pearson et al.’s (2015) initial works on person-centered heterogeneity in mindfulness, a number of other research teams have examined the profiles of mindfulness with different samples of varying age ranges, from adolescents (e.g., Calvete et al., 2020) to older adults (e.g., Zhu et al., 2020). Many of the person-centered papers have focused on college students or adults and derived four mindfulness facet profiles (Pearson et al., 2015; Bravo et al., 2016, 2018; Kimmes et al., 2017; Lam et al., 2018; Ford et al., 2020; Grund et al., 2021; De Souza Marcovski and Miller, 2023; Hémond-Dussault et al., 2023; Hou et al., 2023). In these four-profile findings, *high mindfulness* and *low mindfulness* profiles are homogeneous in nature; the individuals in these profiles score relatively high or relatively low on all facets, respectively. A *non-judgmentally aware* profile is characterized by high scores on the non-judging and acting with awareness facets and low scores on observing. Finally, a *judgmentally observing* profile typically has high scores on observing and low scores on acting with awareness and non-judging (Lecuona et al., 2022; Wang et al., 2022; Carlon et al., 2023). These heterogeneous profiles suggest that there is a meaningful differentiation between individuals who score homogeneously on the mindfulness facets and individuals who display divergent patterns of scores across the facets.

Despite the frequency of these four profiles in person-centered research, there is some variability in the profiles derived across different samples. For example, Zhang et al. (2019) examined Chinese adolescents and derived *judging and high mindfulness, non-reacting observing, low mindfulness, and non-judgmentally aware* profiles. Gu et al. (2020) extracted *low mindfulness, moderate mindfulness, high mindfulness, and non-judgmentally aware* profiles in a sample of age-variant individuals with major depressive disorder in remission. Other papers have derived only three profiles (e.g., Calvete et al., 2020; Bronchain et al., 2021). For instance, in Calvete et al.’s (2020) study with adolescents, *moderate mindfulness, non-judgmentally aware,* and *judgmentally observing* profiles were found. Still other researchers extracted only two profiles such as Gómez-Odriozola and Calvete (2021) and Marques et al. (2020) who examined college students and adults with sleep disturbances. Gómez-Odriozola and Calvete (2021) extracted a profile with high FFMQ scores on all facets expect observing and a profile with low FFMQ scores on all facets except observing, whereas Marques et al. (2020) derived homogeneous high and low mindfulness profiles. Given the inconsistency across studies with different age groups, more research is needed to solidify the understanding of the mindfulness profiles across a large lifespan sample.

An important aspect of the mindfulness profiles is that they differentially relate to well-being, with some profiles seemingly more adaptive than others. As might be expected, the homogeneous mindfulness profiles (i.e., *high mindfulness, moderate mindfulness, low mindfulness*) tend to follow the same pattern of results as variable-centered studies (Lecuona et al., 2022). That is, individuals in the high mindfulness profile have significantly greater well-being as measured by a variety of variables such as happiness, anxiety and depressive symptoms, life satisfaction, and self-regulation than those in the low mindfulness profile (Suh et al., 2022; Wang et al., 2022; De Souza Marcovski and Miller, 2023). In studies where a moderate mindfulness profile was found, individuals in the profile generally had intermediate scores on well-being outcomes (e.g., Calvete et al., 2020; Gu et al., 2020; Zhu et al., 2020; Grund et al., 2021; Leach et al., 2021).

For the most frequently found heterogeneous facet profiles (i.e., *non-judgmentally aware* and *judgmentally observing*), the non-judgmentally aware profile was adaptive and often comparable to the high-mindfulness profile in terms of well-being outcomes, with individuals showing lower depressive and anxiety symptoms (Pearson et al., 2015; Bravo et al., 2016; Gu et al., 2020; Zhu et al., 2020; Bronchain et al., 2021). Conversely, those in the judgmentally observing profile had higher levels of depressive and anxious symptoms than the high mindfulness and non-judgmentally aware profiles (Pearson et al., 2015; Bravo et al., 2016; Leach et al., 2021; De Souza Marcovski and Miller, 2023). Yet, the patterns of results for these heterogeneous profiles are not always unequivocal (Lecuona et al., 2022). For example, Sahdra et al. (2017) found that the non-judgmentally aware profile had nearly identical levels of life satisfaction than the judgmentally observing profile. Given that different mindfulness profiles tend to be distinct in their connections to well-being outcomes, it is important to take a lifespan approach to understand mindfulness profiles, especially since few studies have taken such an approach (e.g., Ford et al., 2020). Both the profiles that exist in a large, lifespan sample of Canadian participants and the distribution of younger and older individuals within these mindfulness profiles is unclear. Thus, we attempted to fill this research gap.

The current research aimed to explore mindfulness profiles in a large sample of age-variable Canadians. Once established, we compared the profiles across well-being outcomes to investigate whether certain profiles were more adaptive than others from a lifespan perspective. Profiles were also compared across age to determine where the majority of youth, adults, and older adults were situated. As recommended for latent profile analyses, we did not have any predetermined hypotheses. However, based on previous findings, we expected that we would find at least high mindfulness and low mindfulness profiles, and potentially two heterogeneous profiles reflecting those who are non-judgmentally aware and judgmentally observing. Moreover, we expected that individuals clustered into the high mindfulness and non-judgmentally aware profiles would report relatively higher scores on most well-being outcomes compared to those in the low mindfulness and judgmentally observing profiles.

## Materials and methods

### Participants and procedure

Canadian participants were recruited through the panel crowdsourcing platform, Qualtrics XM (2023). Data from 1,600 participants (49.8% women) was collected, with a goal for equivalence across age cohorts (14–17, 18–24, 25–34, 35–44, 45–54, 55–64, 65–74, and 75+ years of age) and binary gender (half women). Participants were 45.74 years of age on average (*SD* = 21.09; *range* = 14–90). Most adult participants had completed college or university (49.6%), had an annual household income in the range of $20,000–$80,000 (53.6%), and were not married or in common-law relationships (51.8%). Across the entire sample, participants were Western or Eastern European (50.6%); East Asian (6.5%); South Asian (5.3%); Southeast Asian (3.4%); African (5.3%); Indigenous (4.2%); Central American (3.3%); had multiple ancestral origins (8.5%); and less than 3.0% (per origin) reported South American, Middle Eastern or West Asian, Central Asian, Polynesian or Pacific Islander origins. See Supplementary Table S1 for more in-depth information on participant ancestry. All participants provided informed consent and were paid a nominal and pre-arranged amount by Qualtrics XM (2023) for completion of the study. The complete participant survey, which included variables not reported on in this manuscript, took approximately 15 min to complete. Payment amount was determined by the panel vendor that collected the data, and is not available to report. In line with the Tri-Council Policy Statement: Ethical Conduct for Research Involving Humans (TCPS2) guidelines, the authors’ ethics board determined that parental consent was not required for the adolescent participants given that the study was minimal risk and these individuals could understand the significance of the research and the implications of the risk and benefits to themselves. Ethics approval was obtained from the Simon Fraser University Research Ethics Board (#30001690) on May 23, 2023.

### Measures

#### Mindfulness

Facets of mindfulness were measured using the Five Facet Mindfulness Questionnaire Short Form (FFMQ-SF; Baer et al., 2006; Gu et al., 2016). The 15-item measure assesses the mindfulness facets by three items each: *observing*, how we perceive and attend to the world around us (e.g., I notice how foods and drinks affect my thoughts, bodily sensations, and emotions) *describing*, how we label our internal experiences and express them to others (e.g., I am good at finding words to describe my feelings), *acting with awareness*, attention to our actions with careful thought rather than on automatic (e.g., I find myself doing things without paying attention [reversed]), *non-judging*, self-acceptance and not judging oneself for inner experiences (e.g., I tell myself I should not be feeling the way I’m feeling [reversed]), and *non-reacting*, detaching from inner experiences in order not to react to them negatively (e.g., When I have distressing thoughts or images I am able to just notice them without reacting). Responses indicate frequency of these behaviors on a 5-point Likert scale (1 = never; 5 = always). The original full-length measure as well as shorter versions have showed excellent psychometric properties with adults (Baer et al., 2006) and with children as young as 10 years of age (Cortazar et al., 2020). Reliability as indicated by Cronbach’s alpha (α) and McDonald’s (ω) is depicted in Table 1, alongside correlations among variables. Confirmatory factor analyses of the 15-item FFMQ in the present sample showed excellent factor loadings as mapped onto the same 5 factors as previously validated, with loadings between 0.612 to 0.854 and with all cross-loadings less than 0.326.

**Table 1:** Means, standard deviations, intercorrelations, and reliability of the variables of interest.

Variable	1	2	3	4	5	6	7	8	9	10	11
1. M-AVE	–										
2. M-OB	0.47^***^	–									
3. M-DE	0.74^***^	0.22^***^	–								
4. M-AA	0.64^***^	0.03	0.36^***^	–							
5. M-NJ	0.64^***^	−0.04	0.34^***^	0.46^***^	–						
6. M-NR	0.63^***^	0.30^***^	0.34^***^	0.16^***^	0.20^***^	–					
7. SWL	0.37^***^	0.07^**^	0.25^***^	0.21^***^	0.34^***^	0.27^***^	–				
8. EWB	0.40^***^	0.15^***^	0.31^***^	0.21^***^	0.29^***^	0.32^***^	0.70^***^	–			
9. DASS-D	−0.53^***^	−0.06^*^	−0.36^***^	−0.41^***^	−0.51^***^	−0.32^***^	−0.57^***^	−0.60^***^	–		
10. DASS-A	−0.33^***^	0.10^***^	−0.25^***^	−0.33^***^	−0.39^***^	−0.17^***^	−0.25^***^	−0.26^***^	0.55^***^	–	
11. DASS-S	−0.48^***^	0.04	−0.32^***^	−0.44^***^	−0.48^***^	−0.28^***^	−0.40^***^	−0.38^***^	0.69^***^	0.56^***^	–
*M (SD)*	3.15 (0.53)	3.10 (0.84)	3.05 (0.91)	3.26 (0.82)	3.30 (0.89)	3.05 (0.79)	4.12 (1.43)	3.93 (1.19)	1.86 (0.74)	1.80 (0.63)	2.01 (0.65)
α	0.78	0.60	0.78	0.72	0.74	0.71	0.89	0.83	0.86	0.69	0.78
ω	0.75	0.62	0.79	0.72	0.76	0.71	0.90	0.83	0.86	0.69	0.79

*N* = 1600. M-TOT, Mindfulness Average Score; M-OB, Mindfulness-Observing; M-DE, Mindfulness-Describing; M-AA, Mindfulness-Acting with Awareness; M-NJ, Mindfulness-Non-judging; M-NR, Mindfulness-Non-Reactivity; SWL, Satisfaction with Life; EWB, Existential Well-Being; DASS-D, Depression; DASS-A, Anxiety; DASS-S, Stress.

^*^p < 0.05, ^**^p < 0.01, ^***^p < 0.001.

#### Life satisfaction

Life satisfaction was measured using the 5-item Satisfaction with Life Scale (Diener et al., 1985). Participants were asked to indicate how much they agree with each item (e.g., In most ways my life is close to my ideal) on a 7-point Likert scale (1 = strongly disagree; 7 = strongly agree). Widely used with various age groups, the scale has shown excellent psychometric properties in previous work, including adolescent samples (Neto, 1993; Pavot and Diener, 1993; Jovanović, 2016).

#### Existential well-being

Well-being was measured using the 3-item subscale of Existential Well-being from the Spiritual Well-Being Scale (Bufford et al., 1991; Cotton et al., 2005; Malinakova et al., 2017; Tavel et al., 2022). The Existential Well-Being subscale is a measure of one’s sense of purpose in life. Participants rated their agreement with each item (e.g., I believe there is some real purpose for my life.) on a 7-point Likert scale (1 = strongly disagree; 7 = strongly agree). The measure has shown excellent psychometric properties across various sample groups from adolescents to adults (Malinakova et al., 2017; Paloutzian et al., 2021).

#### Mental health

Mental health was measured using the Depression, Anxiety, and Stress Scale-12 (DASS-12; Osman et al., 2012; Ali et al., 2022), which asked participants to rate their intensity of symptoms over the last week. The 12-item measure assesses three subscales by four items each: depression (e.g., I felt down-hearted and blue), anxiety (e.g., I experienced trembling) and stress (e.g., I found it difficult to relax). Responses are provided on a 4-point Likert scale (1 = did not apply to me at all; 4 = applied to me very much or most of the time). Various longer and shorter forms of the DASS have shown excellent psychometric properties across various age groups spanning from adolescence to older adulthood (Szabó, 2010; Wood et al., 2010).

### Analytic plan

There were no missing data in this study due to the nature of Qualtrics XM (2023) data collection procedures. We conducted latent profile analyses (LPA) to identify profiles of endorsement of the five different mindfulness facets. Latent profile analysis is a data-driven process that determines profiles of responses based on similar patterns of responding while accounting for conditional probabilities of an individual’s membership in each profile. In the present work, standardized FFMQ subscale scores of observing, describing, acting with awareness, non-judging, and non-reacting were used as indicator variables. Gender and age were added as covariates in the model. Models with two to six profiles were examined for model fit. Models were considered better fitting if, compared to models with a different number of profiles, they demonstrated (i) smaller values of the Akaike Information Criterion (AIC), the Bayesian Information Criterion (BIC), and the sample size adjusted BIC (aBIC), (ii) model entropy closer to 1.00, and (iii) a significant Vuong-Lo–Mendell–Rubin Likelihood Ratio Test (VLMR LRT) at *p* < 0.05 (Ferguson et al., 2020; Weller et al., 2020). In addition to general model fit, experts advise that the interpretability of profiles and sufficient sample size representation in the smallest emerging profile are considered in selecting the best fitting model (Nylund-Gibson and Choi, 2018). Sample sizes that are too small (e.g., less than *n* ≈ 100) can indicate groups that are too small to be found in the true population or overextraction of profiles in the case of small sample sizes that lack reliable detection of groups or profiles with rare prevalence (see Tueller and Lubke, 2010; Morgan, 2015). As such, we determined the final model solution largely based on the model fit statistics (AIC, BIC, aBIC, entropy, and VLMR LRT), with further consideration of interpretability and sample sizes of profiles.

Next, profiles derived from the LPA were compared on life satisfaction, existential well-being, and mental health. We used the 3-step method for comparisons (Asparouhov and Muthén, 2014), as this method accounts for the conditional probability of membership across all profiles for each individual when comparing profiles. The 3-step approach provides chi-square (χ^2^) scores for each comparison. A Bonferonni adjustment to respect the *p*-value threshold was applied for multiple comparisons, for example, with significance set at *p* < 0.005 for each comparison for a 5-profile solution.

## Results

For the entire sample, intercorrelations across most of the variables of interest were significant (Table 1). The observing facet was the only exception with non-significant associations found between observing and acting with awareness, *r* = 0.03, *p* = 0.234; non-judging, *r* = −0.04, *p* = 0.142; and stress, *r* = 0.04, *p* = 0.156.

Model fit statistics for the 2- to 6-profile solutions are shown in Table 2. AIC decreased with each added profile, while BIC and aBIC were both optimally lowest for the 5-profile solution and entropy was highest for the 5-profile solution. VLMR LRT was significant at the 2-profile solution, suggesting that exploring data-driven heterogeneity was relevant in our sample. Further, VLMR LRT was significant for the 4- and 5-profile solutions, suggesting that the 5-profile solution fit the data better than the 4-profile solution, which fit better than the 3-profile solution. Although the 6-profile solution had the lowest AIC value, the 5-profile model had lower BIC, aBIC, and higher entropy than the 6-profile model. Moreover, VLMR LRT was not significant for the 6-profile solution implying that this model did not fit the data significantly better than the 5-profile model. The 6-profile model also included at least one profile representing less than 100 individuals, further supporting the selection of the 5-profile solution over the 6-profile option. Taken together, the 5-profile solution was chosen as the best fitting model and further considered for analyses. Average latent profile posterior probabilities ranged from 0.74 to 0.83 across the diagonal for the 5-profile solution.

**Table 2:** Model fit statistics for the 2–6 profile models.

Profiles	AIC	BIC	aBIC	Entropy	Smallest *N*	Smallest ALCP	VLMR
2	21836.118	21932.918	21875.735	0.615	796	0.879	<0.001
3	21602.352	21742.174	21659.577	0.609	309	0.802	0.207
4	21436.896	21619.739	21511.728	0.631	224	0.760	0.004
5	21372.982	21598.848	21465.422	0.671	111	0.739	0.002
6	21362.461	21631.349	21472.508	0.599	93	0.681	0.884

AIC, Aikaike Information Criterion; BIC, Bayesian Information Criterion; aBIC, sample size adjusted BIC; ALCP, Average Latent Class Probability; VLMR, Vuong-Lo–Mendell–Rubin Likelihood Ratio Test.

**Figure 1:**
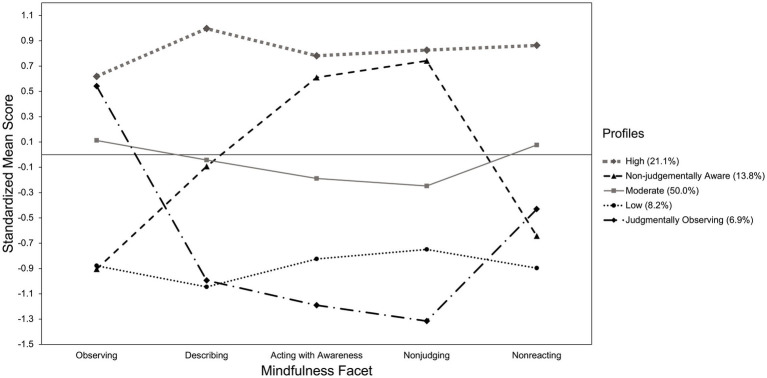
Five profile solution for the five facets of mindfulness. Standard errors ranged from 0.063 to 0.082 in the high mindfulness profile, from 0.059 to 0.080 in the moderate mindfulness profile, from 0.127 to 0.151 in the low mindfulness profile, from 0.094 to 0.139 in the nonjudgmentally aware profile, and from 0.110 to 0.186 in the judgmentally observing profile.

In the 5-profile solution, depicted in Figure 1, the *high mindfulness* profile (*n* = 338; 21.1% of the sample) was marked by high overall mindfulness across the five facets, the *moderate mindfulness* profile (*n* = 800; 50.0% of the sample) by moderate overall mindfulness across the five facets, and the *low mindfulness* profile (*n* = 131; 8.2% of the sample) by low overall mindfulness across most of the facets. A fourth *non-judgmentally aware* profile (*n* = 220; 13.8% of the sample) also emerged, marked by low levels of observing and non-reactivity, moderate levels of describing, and relatively high levels of acting with awareness and non-judging. Finally, the *judgmentally observing* profile (*n* = 111; 6.9% of the sample) was marked by high levels of observing and low levels of non-judging, acting with awareness, and describing.

### Profile comparisons

Profile comparisons are depicted in Table 3. High mindfulness and nonjudgmentally aware profile members were older compared to other profiles, while judgmentally observing profile members were the youngest compared to the rest. Moderate and low mindfulness profile members fell between these groups with regards to age. Additionally, the ratio of women in the judgmentally observing profile was higher than in other profiles. The highest proportions of racialized minorities (or persons of color) were observed in the moderate mindfulness and judgmentally observing profiles. Individuals in the low mindfulness profile were least likely to be married, although this proportion did not significantly differ from the moderate profile. Profiles did not significantly differ in income and education levels.

**Table 3:** Profile comparisons for 5-profile solution.

	*χ*^2^/*F, p*	High mindful	Moderate mindful	Low mindful	Nonjudgmental aware	Judgmentally observing
Demographics						
Age	153.56, <0.001	60.03_a_	41.18_b_	37.81_b_	57.87_a_	20.37_c_
Women (%)	25.57, <0.001	48.8_a_	46.8_a_	52.7_a_	49.5_a_	72.1_b_
Persons of color (%)	73.11, <0.001	36.1_a_	54.9_bc_	49.6_ab_	41.8_a_	65.8_c_
Univ. graduate (%)	8.62, 0.07	54.2_a_	58.8_a_	56.5_a_	48.1_a_	45.8_a_
Married (%)	10.82, 0.03	52.0_a_	49.0_ab_	35.1_b_	54.6_a_	56.3_a_
Income	10.24, 0.04	3.92_a_	4.29_a_	3.71_a_	3.97_a_	3.47_a_
Well-being						
Life satisfaction	197.51, <0.001	4.89_a_	4.03_b_	2.67_c_	4.57_a_	3.48_d_
Existential well-being	205.42, <0.001	4.70_a_	3.90_b_	2.64_c_	3.94_b_	3.60_b_
Depression	559.83, <0.001	1.26_a_	1.97_b_	2.64_c_	1.47_d_	2.66_c_
Anxiety	271.33, <0.001	1.47_a_	1.90_b_	2.00_b_	1.45_a_	2.62_c_
Stress	493.64, <0.001	1.54_a_	2.14_b_	2.57_c_	1.53_a_	2.77_c_

Values in profile columns represent means unless otherwise indicated. Persons of color include the following self-reported ancestral origins: North Africa, Africa, Central and South America, Middle East or West Asia, East Asia, South Asia, Southeast Asia, Central Asia, Polynesia or Pacific Islands, Indigenous groups, and multiple origins. University graduate status (high school graduate) and marital status (married or co-habiting) proportions represent only those adults 23 years of age and older. All χ^2^ represent overall tests with 4 degress of freedom; F statistics are depicted only for age, with 4 between-groups and 1,595 within-groups degrees of freedom. Different subscripts across columns depict significantly different statistics at *p* < 0.005. Conversely, columns that share subscripts depict no significant difference across columns. Take the persons of color row for instance. The frequencies of the high mindful, low mindful, and nonjudgmentally aware profiles do not significantly differ as they all share an _a_ subscript; the frequencies of the low mindful and moderate mindful profiles do not significantly differ as they share a _b_ subscript; and the frequencies of the moderate mindful and judgmentally observing profiles do not significantly differ as they share a _c_ subscript. The low mindful profile has two subscripts, _ab_, because its frequency does not significantly differ from the profiles with an _a_ subscript and moderate mindful with a _b_ subscript. However, the low mindful profile frequency significantly differs from judgmentally observing because these profiles do not share the _c_ subscript.

For the high, moderate, and low mindfulness profiles, results indicated that individuals generally high in mindfulness reported better life satisfaction, existential well-being, and mental health compared to those moderate and low in mindfulness. Individuals both high and moderate across mindfulness facets reported better life satisfaction, existential well-being, and mental health compared to those low in the facets; however, those in the moderate and low mindfulness profiles did not differ in levels of anxiety. Participants from these three homogenous facet profiles composed approximately four-fifths of the overall sample.

**Figure 2:**
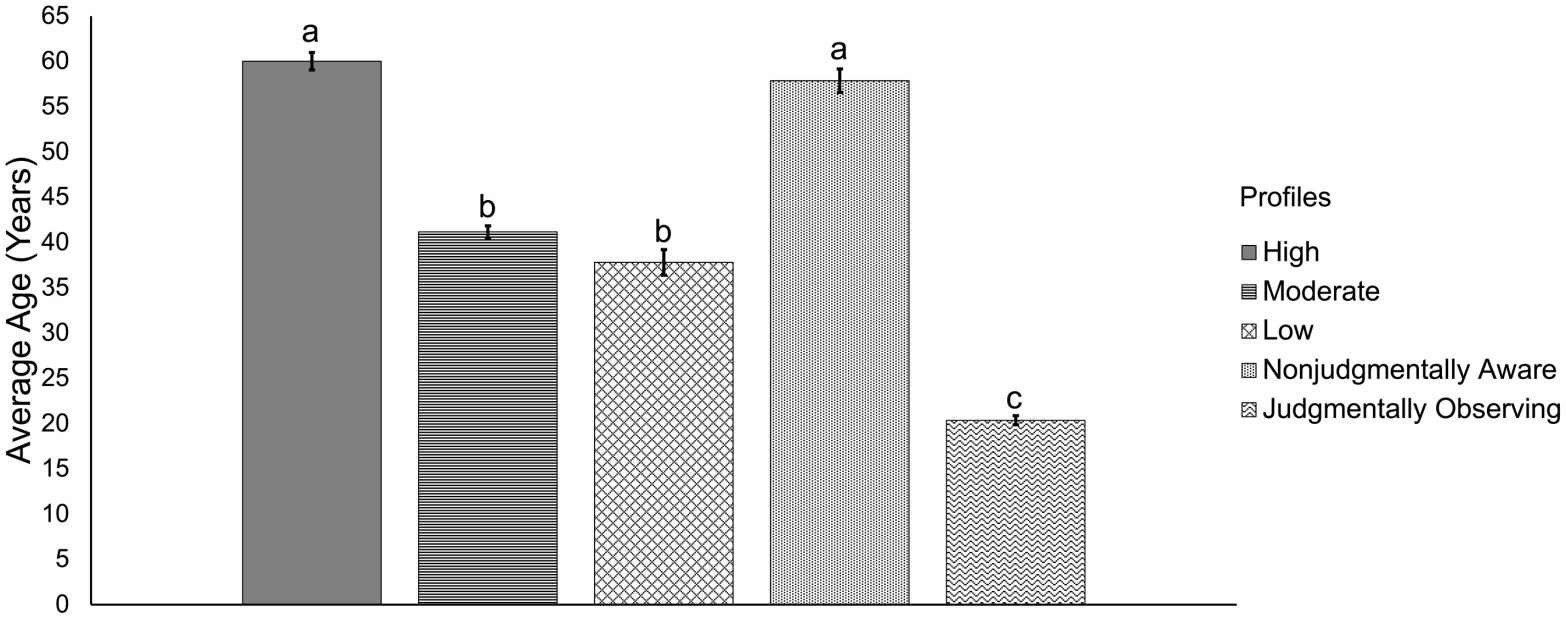
Age across the five mindfulness profiles. Different subscripts depict significantly different means at *p* < 0.005. Conversely, bars that share subscripts depict no significant difference across their means. For example, subscript _a_ occurs across the high and nonjudgmentally aware bars for age and thus, these profiles do not significantly differ in average age. However, the high and nonjudgmentally aware profile do significantly differ from the other three profiles that do not share the _a_ subscript.

**Figure 3:**
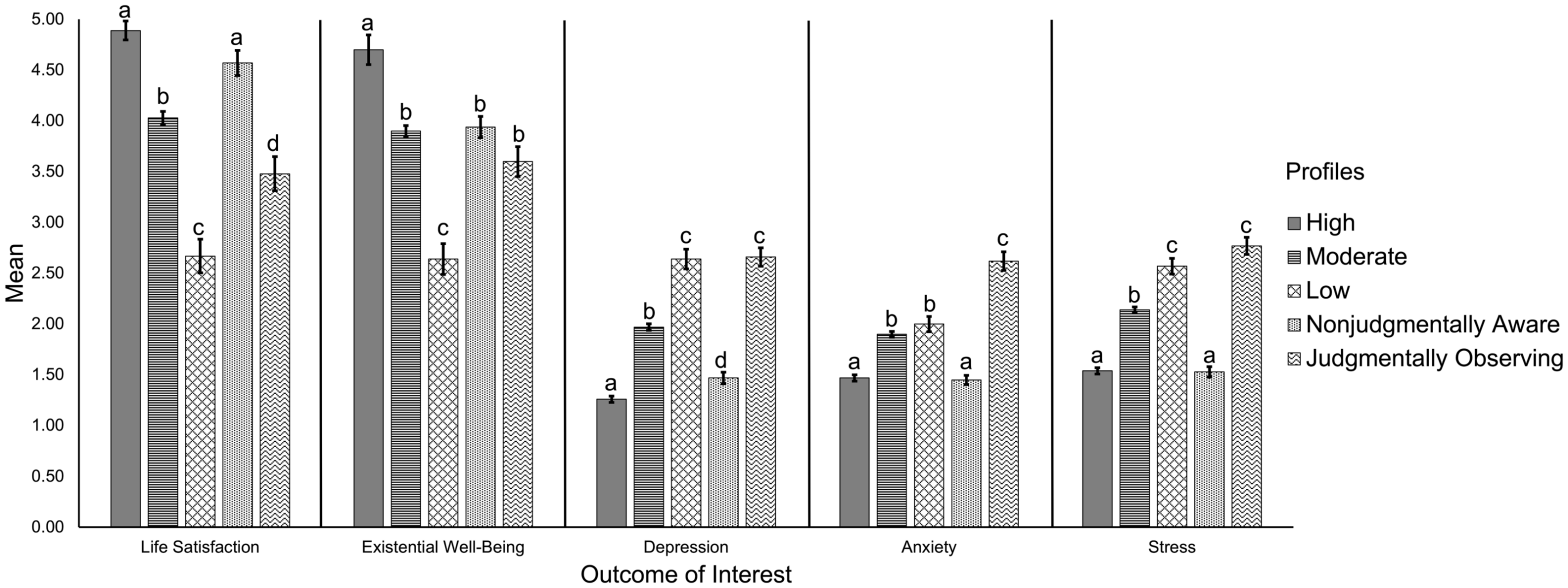
Mental health and well-being across the five mindfulness profiles. Different subscripts within a certain outcome of interest depict significantly different means at *p* < 0.005. Conversely, bars that share subscripts depict no significant difference across their means. For example, subscript _a_ occurs across the high mindfulness and nonjudgmentally aware bars for life satisfaction and thus, these profiles do not significantly differ in life satisfaction scores. However, the high and nonjudgmentally aware profiles do significantly differ from the other three profiles that do not share the _a_ subscript.

Two profiles emerged as heterogeneous in mindfulness facets, composing the remaining one-fifth of the overall sample. Individuals who were nonjudgmentally aware reported similar life satisfaction, stress, and anxiety levels as those in the high mindfulness profile, similar existential well-being as those in the moderate mindfulness profile, and more depressive symptoms compared to the high profile but less compared to the moderate profile. Individuals in the judgmentally observing profile reported the highest levels of anxiety, and similar levels of both depression and stress compared to those low in mindfulness. However, they reported similar levels of well-being as those in the moderate mindfulness profile and fell between the moderate and low mindfulness groups in life satisfaction. For clarity, profile comparisons have been depicted in the form of bar charts for the variables of interest (i.e., age, Figure 2; and life satisfaction, existential well-being, depression, anxiety, and stress; Figure 3).

Overall, those who were nonjudgmentally aware reported many of the same positive outcomes as those in the high mindfulness profile, while individuals who were judgmentally observing reported similar mental health but better well-being in other aspects compared to those in the low mindfulness profile.

## Discussion

In this study, we applied a person-centered approach to determine the profiles of mindfulness in a Canadian lifespan sample and examine how these profiles compared on age, well-being, and mental health. Five mindfulness profiles were identified: *high mindfulness, moderate mindfulness, low mindfulness, nonjudgmentally aware,* and *judgmentally observing*. Those in the high mindfulness and nonjudgmentally aware profiles were generally older, while the judgmentally observing profile consisted of relatively younger individuals. As expected, those in the high mindfulness profile reported the greatest mental health and well-being showing high life satisfaction; existential well-being; and low anxiety, depression, and stress. Those in the nonjudgmentally aware profile reported similar levels of life satisfaction, stress, and anxiety as the high profile. Conversely, those in the low mindfulness and judgmentally observing profiles reported worse mental health than the other profiles, with high anxiety, depression, and stress. The moderate mindfulness profile was situated between these profile groups on the mental health and well-being outcomes.

Our study presents the first person-centered mindfulness study to identify that a five-profile model is best fitting to a large-scale, age-variant dataset. Still, the profiles identified in our data were similar to those found across a myriad of studies and samples (e.g., Pearson et al., 2015; Kimmes et al., 2017; Bravo et al., 2018; Lam et al., 2018; Ford et al., 2020). For example, Ford et al. (2020) identified a *combined* total of five profiles across their two analytic approaches applied to the same sample of 715 American respondents aged 20–88: high, low, and moderate mindfulness; nonjudgmentally aware; and judgmentally observing. Hence, across their two analyses, Ford et al. (2020) produced the same five profiles as our present study. Although our sample had a similar age range as Ford and colleagues’, our sample was more than two times larger, likely allowing us to capture all five profiles in a single analytic approach.

In other person-centered research with smaller sample sizes than the current study (i.e., *N* between 200–900), researchers have typically identified only four profiles (e.g., Bravo et al., 2016, 2018; Kimmes et al., 2017; Lam et al., 2018; Zhang et al., 2019; Ford et al., 2020; Gu et al., 2020; Stanmyre et al., 2022; Wang et al., 2022; Hémond-Dussault et al., 2023). Aside from sample size, differences in the number of extracted profiles may have occurred due to the nature of the unique samples. For instance, Pearson et al. (2015), Bravo et al. (2016), and Kimmes et al. (2017) examined samples of American college students. In these studies, the same four profiles were found: high mindfulness, low mindfulness, nonjudgmentally aware, and judgmentally observing. Gu et al. (2020) sampled adult participants with a history of depression from the United Kingdom and found a low, moderate, and high mindfulness, and a nonjudgmentally aware profile. Whereas Wang et al. (2022) found a moderate and high mindfulness profile and a nonjudgmentally aware and judgmentally observing profile in a sample of cancer patients from the Netherlands. Moreover, different cultural groups may display different profile combinations altogether as Zhang et al. (2019) found judging and high mindfulness, non-reacting observing, non-judging & low mindfulness, and non-judgmentally aware profiles in Chinese adolescents. These studies all used the 39-item version of the FFMQ suggesting that the dissimilarity in findings was likely due to sample differences rather than measurement differences.

Our derivation of five profiles may reflect our sample’s uniqueness, containing Canadians with a wide age range of 14–90 years. Further, a large sample size ensured that enough participants would be represented in each identified profile, including the rare ones. For instance, the judgmentally observing profile made up only 6.9% of the sample but still included over 100 participants. Moreover, with this wide range, all profiles across the lifespan might have been able to emerge. This assertion is plausible given the age variation found across profiles: the high mindfulness and nonjudgmentally aware profile tended to be older, while the judgmentally observing profile tended to be younger. This age variance echoes prior research findings suggesting that older individuals tend to have better developed self-regulation abilities (Geldhof et al., 2017), which in itself has been found to connect to greater mindfulness (Farb et al., 2014). Regarding the nonjudgmentally aware profile, our findings also confirm previous research that suggests that old age is related to present-moment attention and non-judgment specifically (Mahlo and Windsor, 2021). On the other hand, youth and adolescent individuals are more prone to self-judgment (Neff, 2003), and preoccupation on “observing” the thoughts and feelings of others as peer social acceptance becomes very important during this developmental stage (Bowker et al., 2014; Jane-Frances and Ebele, 2014). Thus, our results suggest that the proportion of those characterized by the judgmentally observing profile in the population may skew younger.

One goal of this research was to explore the age distribution of individuals within the mindfulness profiles. For the homogeneous profiles (i.e., high, moderate, and low mindfulness), our results suggest that individuals, on average, may be more mindful as they grow older. Accordingly, younger participants tended to congregate in the low mindfulness profile and the oldest participants clustered in the high mindfulness profile. This pattern of results is consistent with other variable-centered and person-centered studies that have found positive associations between increasing age and greater mindfulness (Hohaus and Spark, 2013; Prakash et al., 2015; Ford et al., 2020; Zhu et al., 2020; Shook et al., 2021; Wang et al., 2022). Aging theory might explain this connection. For instance, the Socioemotional Selectivity Theory suggests that as one grows older, there is heightened awareness that life is time limited. Consequently, the motivation to fully experience and be aware of the present moment increases (Carstensen, 2006; Mahlo and Windsor, 2021). The relationship between age and mindfulness might also have a neurological explanation. The prefrontal cortex plays a key role in mindfulness-related cognitive functions such as self-regulation of thoughts and emotions and self-reflection (i.e., reflecting on the focus of attention and awareness; Zelazo and Lyons, 2012). Furthermore, development of the insula – theorized to maintain the subjective awareness of internal body states—has been found to predict higher mindfulness in adolescents (Friedel et al., 2015). It is known that the prefrontal cortex and the insula continue to develop during adolescence, and therefore, younger individuals may have less of a neuropsychological capacity to be highly mindful.

Regarding the heterogeneous profiles, the nonjudgmentally aware group was similar in age to the high mindfulness profile and relatively older than other profiles. Interestingly, the judgmentally observing profile was significantly younger than all other profiles, including the low mindfulness group. This finding is unique in the context of variable-centered investigations of mindfulness; one might expect to find the youngest individuals in the low mindfulness profile since general low mindfulness has been associated with younger age. Yet, the observing score was higher in the judgmentally observing profile than in the low mindfulness profile. It appears that the quality of observing in the absence of high scores on other facets might be more typical of younger participants. During adolescence, individuals often grapple with their identity and sense of self and there can often be a greater preoccupation on social acceptance and approval (Bowker et al., 2014; Jane-Frances and Ebele, 2014). This preoccupation may be explained by the developmental construct of an *imaginary audience* wherein adolescents feel that others are especially concerned about their appearance and behavior (Elkind, 1967; Alberts et al., 2007). Consequently, younger individuals may devote more attention to external cues and social messages in order to appease the imaginary audience (Jane-Frances and Ebele, 2014). Part of the observing facet of mindfulness involves attention to external experiences. Thus, youth and adolescents may be developmentally predisposed to high levels of observing that mark the judgmentally observing profile. Overall, the nuance provided by the person-centered view of mindfulness created meaningful age differences that are typically not captured by variable-centered analyses.

The second objective of the present study was to examine the differences in well-being and mental health across the mindfulness profiles. Similar to our findings for age, the pattern of results for the homogeneous profiles was consistent with variable-centered studies. That is, individuals in the high mindfulness profile exhibited the highest well-being and mental health, those in the low mindfulness profile reported the lowest levels of both, and those in the moderate mindfulness profile fell in between. These findings align with numerous previous variable-centered findings (Prazak et al., 2012; Alleva et al., 2014; Zimmaro et al., 2016; Mayer et al., 2019; Li et al., 2022).

For the heterogeneous profiles, the nonjudgmentally aware profile generally had comparable levels of mental health and well-being as the high mindfulness group albeit with lower existential well-being and more depressive symptoms. On the other hand, the judgmentally observing profile had a similar mental health and well-being composition as the low mindfulness group. However, this profile had higher anxiety than the low mindfulness profile, suggesting that being judgmentally observant may carry the highest risk of poor well-being and mental health. The pattern of the nonjudgmentally aware profile being similar to the high mindfulness profile and the judgmentally observing profile being similar to the low mindfulness profile has been supported by several profile-approach papers investigating college students (Pearson et al., 2015; Bravo et al., 2016; Bronchain et al., 2021), adults with a history of depression (Gu et al., 2020), and Dutch adults (Zhu et al., 2020).

However, the present findings are at odds with results from prior works by Ford et al. (2020), who found that the nonjudgmentally aware profile was not associated with improved well-being, and Sahdra et al. (2017), who found that the nonjudgmentally aware profile had nearly equal levels of life satisfaction as the judgmentally observing profile. We pose that the unique findings of these two works are due to methodological differences. Our findings, reflecting similar findings as the large majority of smaller sample-size studies, utilized the 3-step method (Asparouhov and Muthén, 2014) to compare the profiles across our mental health and well-being variables. This method is the current gold-standard technique for LPA profile comparisons as it supports conditional probabilities of profile membership for each individual within the comparisons. The method provides a better estimate of differences across profiles than techniques that assign individuals to profiles in a discreet manner, better supporting our own and the majority of findings regarding profile differences on mental health and well-being.

The current results imply that the heterogeneity of mindfulness should be considered in the context of mindfulness, well-being, and mental health across the lifespan. It appears as though certain combinations of mindfulness facets are more adaptive than others. For example, acting with awareness, when paired with non-judging, might be sufficient to support mental health flourishing; observing, describing, and non-reacting may not independently provide the same benefits as having high scores on all of the facets. On the other hand, observing alone appears to undermine mental health and well-being, particularly when all other facets are low.

These findings are crucial to support further research and practice relating to mindfulness-based interventions. Since the judgmentally aware profile was associated with greater mental health and well-being, it may be useful to focus on the non-judging and acting with awareness facets in a time-sensitive or resource-limited intervention (i.e., with minimal budgets or few sessions). Future researchers may consider taking an existing mindfulness intervention that addresses all facets of mindfulness and reduce it to specifically focus on non-judgment and acting with awareness. Then, the researchers could examine whether participant mental health outcomes of the revised intervention are comparable to the lengthier, full-facet, original version. Furthermore, in clinical contexts, if the observing facet appears to be independently elevated in certain individuals, then ensuring its combination with other facets such as acting with awareness and non-judging may provide targeted support for adaptive functioning. Applied clinical researchers may elect to provide such targeted support to members of the judgmentally observing profile to assess its efficacy in bolstering their mental health. Additional research is required to adequately address these propositions, especially in intervention settings. Still, experimental person-centered investigations of mindfulness provide essential nuance into our understanding of the mindfulness to well-being and mental health connections that mindfulness-based interventions should consider.

The present study was not without limitations. Although the sample was counter-balanced by age-cohort and binary gender, it was not very diverse and could be characterized as generally Western, educated, industrialized, rich, and democratic (WEIRD; Henrich et al., 2010). Many studies in Western psychology are conducted with WEIRD samples, which has been a common critique throughout the field. As Canada continues to grow in cultural diversity, WEIRD samples may become further non-representative of the greater population. This study used an age-diverse community-based sample, which avoided the other critique of psychology studies generally using first-year university sample pools. Yet, given that the recruitment occurred on Qualtrics XM (2023), those who have access to technology and the means to complete studies on Qualtrics were overrepresented. Future studies may elect to mail out questionnaires to participants to address this concern. With the mindfulness profiles identified across the lifespan in the present sample, future research should attempt to replicate these findings with ethnically diverse and nationally representative samples. Such replications could be conducted using quota sampling techniques with participant quotas derived from Canadian census data regarding key demographic variables (e.g., ethnicity, income, education).

Another limitation is that some of the profiles were comprised of relatively lower percentages of the overall sample (e.g., low mindfulness, 8.2%; judgmentally observing, 6.9%). This raises potential concerns about the replicability and representation of these profiles in the population. However, Nylund-Gibson and Choi (2018) have argued that profiles with 5% or more of the sample are valid given total sample sizes of more than *N* = 1000. Further, all of the five profiles identified in this study had at least 100 participants, suggesting its relevance for a sizable proportion of our sample. Since certain profiles appear to be rarer across the lifespan, it is critical for future person-centered studies utilizing LPA to recruit sufficiently large samples to generate reliable profiles, as smaller samples can mask valid, important, and relevant profiles that are hard to detect due to low prevalence (Masyn, 2013).

The brevity of the scales utilized in the present study was an additional limitation. Short forms of the scales of interest were selected in the interest of the participants, to decrease fatigue effects in completing a long questionnaire. However, some scholars have argued that there are consequences for reliability when short form measures are employed (Smith et al., 2000). For example, using short forms increases the random measurement error within individuals’ response data. Validity can also be potentially compromised when short forms are used. Despite this concern, we derived five mindfulness profiles in this study; all of which had collectively been derived in previous research (Pearson et al., 2015; Kimmes et al., 2017; Bravo et al., 2018; Lam et al., 2018; Ford et al., 2020). In any case, the current findings should be replicated using full-length measures to ensure the robustness of the results.

A final limitation was that participants’ meditation experience was not measured in our study, and as such was not accounted for or compared across profiles. Meditation is a technique that can cultivate mindfulness and previous research has found that the connections between the facets of mindfulness and well-being can differ between meditators and non-meditators (Lilja et al., 2013; Sahdra et al., 2017). For instance, Sahdra et al. (2017) found that participants with meditation experience were more likely to belong to profiles with higher levels of mindfulness. In the present, life-span sample, meditation experience may have varied across age groups. Previous research has shown that older individuals are more likely to have more experience with meditation than younger individuals (e.g., Bergomi et al., 2015; Fuochi and Voci, 2020). Therefore, although age was controlled in the present study’s analyses, meditation experience may have confounded the connection between age and mindfulness. Meditation experience should be controlled in future person-centered examinations of mindfulness and well-being.

The present findings were cross-sectional in nature and could only take a “snapshot” of the mindfulness profiles that may exist across the lifespan in Canada. Applying person-centered approaches to mindfulness is relatively new; the first study of this sort was conducted in 2013 (Lilja et al., 2013). Consequently, there is little historical data to compare against to predict how profiles may *change* over time within a given society. Future work may use analytical techniques such as latent transition models—longitudinal extensions of LPA (Abarda et al., 2020)—to address this research gap. Latent transition models would allow researchers to describe how mindfulness profiles change over time and examine the probability that particular individuals remain in certain profiles.

To conclude, the present study found five profiles of mindfulness in a lifespan sample: high mindfulness, moderate mindfulness, low mindfulness, nonjudgmentally aware, and judgmentally observing. The high mindfulness and nonjudgmentally aware profiles were older and more well-adjusted in terms of mental health and well-being, whereas the low mindfulness and judgmentally observing profiles were the least adjusted and were younger. Our findings account for the heterogeneity in mindfulness and emphasize the importance of taking person-centered approaches to examining the connections between mindfulness and well-being across the lifespan.

## Data availability statement

The raw data supporting the conclusions of this article will be made available by the authors, without undue reservation.

## Ethics statement

The studies involving humans were approved by Simon Fraser University Research Ethics Board. The studies were conducted in accordance with the local legislation and institutional requirements. The participants provided their written informed consent to participate in this study.

## Author contributions

NJ: Conceptualization, Data curation, Investigation, Methodology, Writing – original draft, Writing – review & editing, Visualization. RS: Writing – review & editing. HK: Writing – review & editing, Conceptualization, Data curation, Formal analysis, Funding acquisition, Investigation, Methodology, Supervision, Writing – original draft.

## References

[ref1] Abarda A. Dakkon M. Azhari M. Zaaloul A. Khabouze M. (2020). Latent transition analysis (LTA): a method for identifying differences in longitudinal change among unobserved groups. Procedia Comput. Sci. 170, 1116–1121. doi: 10.1016/j.procs.2020.03.059

[ref2] Alberts A. Elkind D. Ginsberg S. (2007). The personal fable and risk-taking in early adolescence. J. Youth Adolesc. 36, 71–76. doi: 10.1007/s10964-006-9144-4

[ref3] Ali A. M. Alameri R. A. Hendawy A. O. Al-Amer R. Shahrour G. Ali E. M.. (2022). Psychometric evaluation of the depression anxiety stress scale 8-items (DASS-8)/DASS-12/DASS-21 among family caregivers of patients with dementia. Front. Public Health 10:1012311. doi: 10.3389/fpubh.2022.1012311, PMID: 36388286 PMC9641276

[ref4] Alleva J. Roelofs J. Voncken M. Meevissen Y. Alberts H. (2014). On the relation between mindfulness and depressive symptoms: rumination as a possible mediator. Mindfulness 5, 72–79. doi: 10.1007/s12671-012-0153-y

[ref5] Asparouhov T. Muthén B. (2014). Auxiliary variables in mixture modeling: three-step approaches using Mplus. Struct. Equ. Modeling 21, 329–341. doi: 10.1080/10705511.2014.915181

[ref6] Baer R. A. Smith G. T. Hopkins J. Krietemeyer J. Toney L. (2006). Using self-report assessment methods to explore facets of mindfulness. Assessment 13, 27–45. doi: 10.1177/1073191105283504, PMID: 16443717

[ref7] Baer R. A. Smith G. T. Lykins E. Button D. Krietemeyer J. Sauer S.. (2008). Construct validity of the five facet mindfulness questionnaire in meditating and nonmeditating samples. Assessment 15, 329–342. doi: 10.1177/1073191107313003, PMID: 18310597

[ref8] Bergomi C. Tschacher W. Kupper Z. (2015). Meditation practice and self-reported mindfulness: a cross-sectional investigation of meditators and non-meditators using the comprehensive inventory of mindfulness experiences (CHIME). Mindfulness 6, 1411–1421. doi: 10.1007/s12671-015-0415-6

[ref9] Bishop S. R. Lau M. Shapiro S. Carlson L. Anderson N. D. Carmody J.. (2004). Mindfulness: a proposed operational definition. Clin. Psychol. Sci. Pract. 11, 230–241. doi: 10.1093/clipsy.bph077

[ref10] Bowker J. C. Adams R. E. Fredstrom B. K. Gilman R. (2014). Experiences of being ignored by peers during late adolescence: linkages to psychological maladjustment. Merrill-Palmer Q. 60, 328–354. doi: 10.13110/merrpalmquar1982.60.3.0328

[ref11] Bravo A. J. Boothe L. G. Pearson M. R. (2016). Getting personal with mindfulness: a latent profile analysis of mindfulness and psychological outcomes. Mindfulness 7, 420–432. doi: 10.1007/s12671-015-0459-7

[ref12] Bravo A. J. Pearson M. R. Kelley M. L. (2018). Mindfulness and psychological health outcomes: a latent profile analysis among military personnel and college students. Mindfulness 9, 258–270. doi: 10.1007/s12671-017-0771-5, PMID: 29430258 PMC5800780

[ref13] Bronchain J. Raynal P. Chabrol H. (2021). Dispositional mindfulness profiles and cannabis use in young adults. J. Ration. Emot. Cogn. Behav. Ther. 39, 509–521. doi: 10.1007/s10942-020-00382-z

[ref14] Bufford R. K. Paloutzian R. F. Ellison C. W. (1991). Norms for the spiritual well-being scale. J. Psychol. Theol. 19, 56–70. doi: 10.1177/009164719101900106

[ref15] Calvete E. Fernández-González L. Echezarraga A. Orue I. (2020). Dispositional mindfulness profiles in adolescents and their associations with psychological functioning and hypothalamic–pituitary–adrenal axis hormones. J. Youth Adolesc. 49, 1406–1419. doi: 10.1007/s10964-019-01128-6, PMID: 31631232

[ref16] Campos D. Cebolla A. Quero S. Bretón-López J. Botella C. Soler J.. (2016). Meditation and happiness: mindfulness and self-compassion may mediate the meditation–happiness relationship. Pers. Individ. Differ. 93, 80–85. doi: 10.1016/j.paid.2015.08.040

[ref17] Carlon H. A. Earnest J. Hurlocker M. C. (2023). Is mindfulness associated with safer cannabis use? A latent profile analysis of dispositional mindfulness among college students who use cannabis. Mindfulness 14, 797–807. doi: 10.1007/s12671-023-02110-x, PMID: 37997576 PMC10664800

[ref18] Carpenter J. K. Conroy K. Gomez A. F. Curren L. C. Hofmann S. G. (2019). The relationship between trait mindfulness and affective symptoms: a meta-analysis of the five facet mindfulness questionnaire (FFMQ). Clin. Psychol. Rev. 74:101785. doi: 10.1016/j.cpr.2019.101785, PMID: 31751877 PMC6878205

[ref19] Carstensen L. L. (2006). The influence of a sense of time on human development. Science 312, 1913–1915. doi: 10.1126/science.1127488, PMID: 16809530 PMC2790864

[ref20] Cortazar N. Calvete E. Fernández-González L. Orue I. (2020). Development of a short form of the five facet mindfulness questionnaire–adolescents for children and adolescents. J. Pers. Assess. 102, 641–652. doi: 10.1080/00223891.2019.1616206, PMID: 31166802

[ref21] Cotton S. Larkin E. Hoopes A. Cromer B. A. Rosenthal S. L. (2005). The impact of adolescent spirituality on depressive symptoms and health risk behaviors. J. Adolesc. Health 36:529. doi: 10.1016/j.jadohealth.2004.07.017, PMID: 15909358

[ref22] De Souza Marcovski F. C. Miller L. J. (2023). A latent profile analysis of the five facets of mindfulness in a U.S. adult sample: spiritual and psychological differences among four profiles. Curr. Psychol. 42, 14223–14236. doi: 10.1007/s12144-021-02546-1

[ref23] Diener E. Emmons R. A. Larsen R. J. Griffin S. (1985). The satisfaction with life scale. J. Pers. Assess. 49, 71–75. doi: 10.1207/s15327752jpa4901 16367493

[ref24] Elkind D. (1967). Egocentrism in adolescence. Child Dev. 38, 1025–1034. doi: 10.2307/1127100 5583052

[ref25] Farb N. Anderson A. Irving J. Segal Z. V. (2014). “Mindfulness interventions and emotion regulation” in Handbook of emotion regulation. ed. Gross J. J. (New York City, NY: The Guilford Press), 548–567.

[ref26] Ferguson S. L. Moore E. W. G. Hull D. M. (2020). Finding latent groups in observed data: a primer on latent profile analysis in Mplus for applied researchers. Int. J. Behav. Dev. 44, 458–468. doi: 10.1177/0165025419881721

[ref27] Ford C. G. Wilson J. M. Altman N. Strough J. Shook N. J. (2020). Profiles of mindfulness across adulthood. Mindfulness 11, 1557–1569. doi: 10.1007/s12671-020-01372-z

[ref28] Friedel S. Whittle S. L. Vijayakumar N. Simmons J. G. Byrne M. L. Schwartz O. S.. (2015). Dispositional mindfulness is predicted by structural development of the insula during late adolescence. Dev. Cogn. Neurosci. 14, 62–70. doi: 10.1016/j.dcn.2015.07.001, PMID: 26209810 PMC6989825

[ref29] Fuochi G. Voci A. (2020). A deeper look at the relationship between dispositional mindfulness and empathy: meditation experience as a moderator and dereification processes as mediators. Pers. Individ. Differ. 165:110122. doi: 10.1016/j.paid.2020.110122

[ref30] Geiger P. J. Boggero I. A. Brake C. A. Caldera C. A. Combs H. L. Peters J. R.. (2016). Mindfulness-based interventions for older adults: a review of the effects on physical and emotional well-being. Mindfulness 7, 296–307. doi: 10.1007/s12671-015-0444-1 27200109 PMC4868399

[ref31] Geldhof G. J. Fenn M. L. Finders J. K. (2017). “A self-determination perspective on self-regulation across the life span” in Development of self-determination through the life-course. eds. Wehmeyer M. L. Shogren K. A. Little T. D. Lopez S. J. (Dordrecht: Springer), 221–235.

[ref32] Gethin R. (2015). “Buddhist conceptualizations of mindfulness” in Handbook of mindfulness: theory, research, and practice. eds. Creswell J. D. Ryan R. M. Brown K. W. (New York City, NY: The Guilford Press), 9–41.

[ref33] Gómez-Odriozola J. Calvete E. (2021). The role of dispositional mindfulness profiles as predictors of sleep problems through rumination in adolescents over time. Pers. Individ. Differ. 180:110966. doi: 10.1016/j.paid.2021.110966

[ref34] Grund A. Senker K. Dietrich J. Fries S. Galla B. M. (2021). The comprehensive mindfulness experience: a typological approach to the potential benefits of mindfulness for dealing with motivational conflicts. Motiv. Sci. 7, 410–423. doi: 10.1037/mot0000239

[ref35] Gu J. Karl A. Baer R. Strauss C. Barnhofer T. Crane C. (2020). Latent profile analysis of the five facet mindfulness questionnaire in a sample with a history of recurrent depression. Assessment 27, 149–163. doi: 10.1177/1073191117715114, PMID: 28629232 PMC6906539

[ref36] Gu J. Strauss C. Crane C. Barnhofer T. Karl A. Cavanagh K.. (2016). Examining the factor structure of the 39-item and 15-item versions of the five facet mindfulness questionnaire before and after mindfulness-based cognitive therapy for people with recurrent depression. Psychol. Assess. 28, 791–802. doi: 10.1037/pas0000263, PMID: 27078186 PMC4928699

[ref37] Hémond-Dussault V. Dussault É. Hébert M. Godbout N. (2023). Childhood interpersonal trauma and relationality among profiles of mindfulness facets. Mindfulness 14, 348–359. doi: 10.1007/s12671-022-02038-8

[ref38] Henrich J. Heine S. J. Norenzayan A. (2010). Most people are not WEIRD. Nature 466:29. doi: 10.1038/466029a 20595995

[ref39] Hohaus L. C. Spark J. (2013). Getting better with age: do mindfulness and psychological well-being improve in old age? Eur. Psychiatry 28:1. doi: 10.1016/S0924-9338(13)77295-X21920709

[ref40] Hou Y. Zhang Y. Liu Y. Yuan H. Lei X. (2023). Mindfulness profiles among Chinese university students: exploring differences in phenomenon, cognition, and performance of mind wandering. Mindfulness 14, 908–918. doi: 10.1007/s12671-023-02080-0

[ref41] Jane-Frances A. Ebele I. (2014). Assessment of negative self-image and fear of negative evaluation among adolescents and young adults. J. Psychol. Res. 4, 905–914. doi: 10.17265/2159-5542/2014.11.008

[ref42] Jones A. Hook M. Podduturi P. McKeen H. Beitzell E. Liss M. (2022). Mindfulness as a mediator in the relationship between social media engagement and depression in young adults. Pers. Individ. Differ. 185:111284. doi: 10.1016/j.paid.2021.111284

[ref9001] Jovanović V. (2016). The validity of the Satisfaction with Life Scale in adolescents and a comparison with single-item life satisfaction measures: A preliminary study. Qual. Life Res. 25, 3173–3180. doi: 10.1007/s11136-016-1331-5 27262574

[ref43] Kabat-Zinn J. (2003). Mindfulness-based interventions in context: past, present, and future. Clin. Psychol. Sci. Pract. 10, 144–156. doi: 10.1093/clipsy.bpg016

[ref44] Kimmes J. G. Durtschi J. A. Fincham F. D. (2017). Perception in romantic relationships: a latent profile analysis of trait mindfulness in relation to attachment and attributions. Mindfulness 8, 1328–1338. doi: 10.1007/s12671-017-0708-z

[ref45] Lam K. F. Y. Lim H. A. Kua E. H. Griva K. Mahendran R. (2018). Mindfulness and cancer patients’ emotional states: a latent profile analysis among newly diagnosed cancer patients. Mindfulness 9, 521–533. doi: 10.1007/s12671-017-0794-y

[ref46] Leach S. M. Mitchell A. M. Salmon P. Sephton S. E. (2021). Mindfulness, self-reported health, and cortisol: a latent profile analysis. J. Health Psychol. 26, 2719–2729. doi: 10.1177/1359105320931184, PMID: 32508170

[ref47] Lecuona O. García-Rubio C. de Rivas S. Moreno-Jiménez J. E. Rodríguez-Carvajal R. (2022). Unraveling heterogeneities in mindfulness profiles: a review and latent profile analysis of the five facet mindfulness questionnaire short-form (FFMQ-SF) in the Spanish population. Mindfulness 13, 2031–2046. doi: 10.1007/s12671-022-01939-y

[ref48] Li X. Ma L. Li Q. (2022). How mindfulness affects life satisfaction: based on the mindfulness-to-meaning theory. Front. Psychol. 13:887940. doi: 10.3389/fpsyg.2022.887940, PMID: 35846723 PMC9282043

[ref49] Lilja J. L. Lundh L.-G. Josefsson T. Falkenström F. (2013). Observing as an essential facet of mindfulness: a comparison of FFMQ patterns in meditating and non-meditating individuals. Mindfulness 4, 203–212. doi: 10.1007/s12671-012-0111-8

[ref50] Mahlo L. Windsor T. D. (2021). Older and more mindful? Age differences in mindfulness components and well-being. Aging Ment. Health 25, 1320–1331. doi: 10.1080/13607863.2020.1734915, PMID: 32114803

[ref51] Malinakova K. Kopcakova J. Kolarcik P. Geckova A. M. Solcova I. P. Husek V.. (2017). The spiritual well-being scale: psychometric evaluation of the shortened version in Czech adolescents. J. Relig. Health 56, 697–705. doi: 10.1007/s10943-016-0318-4, PMID: 27787695 PMC5320003

[ref52] Marques D. R. Gomes A. A. Pereira A. S. (2020). Mindfulness profiles in a sample of self-reported sleep disturbance individuals. J. Contextual Behav. Sci. 15, 219–224. doi: 10.1016/j.jcbs.2020.01.008

[ref53] Masyn K. E. (2013). “Latent class analysis and finite mixture modeling” in The Oxford handbook of quantitative methods: Statistical analysis, vol. 2. ed. Little T. D. (Oxford: Oxford Press), 551–611.

[ref54] Mattes J. (2019). Systematic review and meta-analysis of correlates of FFMQ mindfulness facets. Front. Psychol. 10, 1–18. doi: 10.3389/fpsyg.2019.02684 31866899 PMC6909938

[ref55] Mayer B. Polak M. G. Remmerswaal D. (2019). Mindfulness, interpretation bias, and levels of anxiety and depression: two mediation studies. Mindfulness 10, 55–65. doi: 10.1007/s12671-018-0946-8, PMID: 30662572 PMC6320741

[ref56] Morgan G. B. (2015). Mixed mode latent class analysis: an examination of fit index performance for classification. Struct. Equ. Modeling 22, 76–86. doi: 10.1080/10705511.2014.935751

[ref57] Neff K. (2003). Self-compassion: an alternative conceptualization of a healthy attitude toward oneself. Self Identity 2, 85–101. doi: 10.1080/15298860309032

[ref58] Neto F. (1993). The satisfaction with life scale: psychometrics properties in an adolescent sample. J. Youth Adolesc. 22, 125–134. doi: 10.1007/BF01536648

[ref59] Nylund-Gibson K. Choi A. Y. (2018). Ten frequently asked questions about latent class analysis. Transl. Issues Psychol. Sci. 4, 440–461. doi: 10.1037/tps0000176

[ref60] Osman A. Wong J. L. Bagge C. L. Freedenthal S. Gutierrez P. M. Lozano G. (2012). The depression anxiety stress Scales-21 (DASS-21): further examination of dimensions, scale reliability, and correlates. J. Clin. Psychol. 68, 1322–1338. doi: 10.1002/jclp.21908, PMID: 22930477

[ref61] Paloutzian R. F. Agilkaya-Sahin Z. Bruce K. C. Kvande M. N. Malinakova K. Marques L. F.. (2021). “The spiritual well-being scale (SWBS): cross-cultural assessment across 5 continents, 10 languages, and 300 studies” in Assessing spirituality in a diverse world. eds. Ai A. L. Wink P. Paloutzian R. F. Harris K. A. (Cham: Springer International Publishing), 413–444.

[ref62] Pavot W. Diener E. (1993). Review of the satisfaction with life scale. Psychol. Assess. 5, 164–172. doi: 10.1037/1040-3590.5.2.164

[ref63] Pearson M. R. Lawless A. K. Brown D. B. Bravo A. J. (2015). Mindfulness and emotional outcomes: identifying subgroups of college students using latent profile analysis. Pers. Individ. Differ. 76, 33–38. doi: 10.1016/j.paid.2014.11.009, PMID: 25530649 PMC4269250

[ref64] Prakash R. S. Hussain M. A. Schirda B. (2015). The role of emotion regulation and cognitive control in the association between mindfulness disposition and stress. Psychol. Aging 30, 160–171. doi: 10.1037/a0038544, PMID: 25545683

[ref65] Prazak M. Critelli J. Martin L. Miranda V. Purdum M. Powers C. (2012). Mindfulness and its role in physical and psychological health. Appl. Psychol. Health Well-Being 4, 91–105. doi: 10.1111/j.1758-0854.2011.01063.x 26286972

[ref66] Qualtrics XM. (2023). Available at: (https://www.qualtrics.com).

[ref67] Reangsing C. Punsuwun S. Schneider J. K. (2021). Effects of mindfulness interventions on depressive symptoms in adolescents: a meta-analysis. Int. J. Nurs. Stud. 115:103848. doi: 10.1016/j.ijnurstu.2020.103848, PMID: 33383273

[ref68] Royuela-Colomer E. Calvete E. (2016). Mindfulness facets and depression in adolescents: rumination as a mediator. Mindfulness 7, 1092–1102. doi: 10.1007/s12671-016-0547-3

[ref69] Sahdra B. K. Ciarrochi J. Parker P. D. Basarkod G. Bradshaw E. L. Baer R. (2017). Are people mindful in different ways? Disentangling the quantity and quality of mindfulness in latent profiles and exploring their links to mental health and life effectiveness. Eur. J. Personal. 31, 347–365. doi: 10.1002/per.2108

[ref70] Shook N. J. Delaney R. K. Strough J. Wilson J. M. Sevi B. Altman N. (2021). Playing it safe: dispositional mindfulness partially accounts for age differences in health and safety risk-taking propensity. Curr. Psychol. 40, 2142–2152. doi: 10.1007/s12144-019-0137-3

[ref71] Smith G. T. McCarthy D. M. Anderson K. G. (2000). On the sins of short-form development. Psychol. Assess. 12, 102–111. doi: 10.1037/1040-3590.12.1.102, PMID: 10752369

[ref72] Stanmyre J. F. Mills D. J. Anthony W. L. Nower L. (2022). Mindfulness profiles among gamblers: exploring differences in gambling behaviors, motivations, cognitions, and mental health. Mindfulness 13, 339–350. doi: 10.1007/s12671-021-01791-6

[ref73] Suh H. Kim S. Y. McCabe E. A. (2022). Profiles of mindfulness and difficulties in emotion regulation and links to work–family–school conflict. J. Am. Coll. Heal. 70, 420–427. doi: 10.1080/07448481.2020.1752696, PMID: 32407176

[ref74] Szabó M. (2010). The short version of the depression anxiety stress scales (DASS-21): factor structure in a young adolescent sample. J. Adolesc. 33, 1–8. doi: 10.1016/j.adolescence.2009.05.014, PMID: 19560196

[ref75] Tavel P. Jozefiakova B. Telicak P. Furstova J. Puza M. Kascakova N. (2022). Psychometric analysis of the shortened version of the spiritual well-being scale on the Slovak population (SWBS-Sk). Int. J. Environ. Res. Public Health 19, 1–12. doi: 10.3390/ijerph19010511, PMID: 35010770 PMC8744853

[ref76] Tomlinson E. R. Yousaf O. Vittersø A. D. Jones L. (2018). Dispositional mindfulness and psychological health: a systematic review. Mindfulness 9, 23–43. doi: 10.1007/s12671-017-0762-6, PMID: 29387263 PMC5770488

[ref77] Tueller S. Lubke G. (2010). Evaluation of structural equation mixture models: parameter estimates and correct class assignment. Struct. Equ. Modeling 17, 165–192. doi: 10.1080/10705511003659318, PMID: 20582328 PMC2890304

[ref79] Wang J. Wei L. Zhu L. Schroevers M. J. (2022). Profiles of mindfulness in cancer patients and associations with psychological outcomes and coping strategies: a person-centered approach. J. Clin. Psychol. 78, 2470–2483. doi: 10.1002/jclp.23346, PMID: 35315081

[ref80] Weller B. E. Bowen N. K. Faubert S. J. (2020). Latent class analysis: a guide to best practice. J. Black Psychol. 46, 287–311. doi: 10.1177/0095798420930932

[ref81] Wood B. M. Nicholas M. K. Blyth F. Asghari A. Gibson S. (2010). The utility of the short version of the depression anxiety stress scales (DASS-21) in elderly patients with persistent pain: does age make a difference? Pain Med. 11, 1780–1790. doi: 10.1111/j.1526-4637.2010.01005.x, PMID: 21134119

[ref82] Zelazo P. D. Lyons K. E. (2012). The potential benefits of mindfulness training in early childhood: a developmental social cognitive neuroscience perspective. Child Dev. Perspect. 6, 154–160. doi: 10.1111/j.1750-8606.2012.00241.x

[ref83] Zhang J. Deng X. Huang L. Zeng H. Wang L. Wen P. (2019). Profile of trait mindfulness and its association with emotional regulation for early adolescents. Pers. Individ. Differ. 147, 12–17. doi: 10.1016/j.paid.2019.04.008

[ref84] Zhu L. Wang J. Schroevers M. J. (2020). Looking beyond the value of individual facets of mindfulness: a person-centered examination of mindfulness. Mindfulness 11, 2349–2359. doi: 10.1007/s12671-020-01452-0

[ref85] Zimmaro L. A. Salmon P. Naidu H. Rowe J. Phillips K. Rebholz W. N.. (2016). Association of dispositional mindfulness with stress, cortisol, and well-being among university undergraduate students. Mindfulness 7, 874–885. doi: 10.1007/s12671-016-0526-8

[ref86] Zoogman S. Goldberg S. B. Hoyt W. T. Miller L. (2015). Mindfulness interventions with youth: a meta-analysis. Mindfulness 6, 290–302. doi: 10.1007/s12671-013-0260-4

