# Supplementary material for: Not all mindfulness is equal: certain facets of mindfulness have important implications for well-being and mental health across the lifespan
Source: Front Psychol. 2024 Apr 15;15:1347487. doi: 10.3389/fpsyg.2024.1347487 (PMC11057496; doi:10.3389/fpsyg.2024.1347487)
Supplement: Supplementary file 1 [file Data_Sheet_1.DOCX]

**Additional Information Regarding Participant Ancestry**

In our paper, we noted the ancestry of our sample. We wish to honor all of our participants’ ancestral origins and thus, this supplementary document provides more in-depth information regarding the breakdown of participant ancestry.

The participants had the following ancestry choices in their questionnaire:

- North Africa
- Africa (not including North African countries)
- Central America
- South America
- Eastern Europe
- Western Europe
- Middle East or West Asia
- East Asia
- Central Asia
- South Asia
- Southeast Asia
- Polynesia or Pacific Islands
- Indigenous groups
- Multiple origins (specify)
- Other (specify)

See Table S1 below for a breakdown of participants’ selections for ancestry across the entire sample.

**Table S1**

*Breakdown of participant ancestry across the entire sample.*

| Ancestry | Number | Percentage of sample (%) |
| --- | --- | --- |
| Africa | 84 | 5.3 |
| Central America | 52 | 3.3 |
| South America | 29 | 1.8 |
| Eastern Europe | 238 | 14.9 |
| Western Europe | 571 | 35.7 |
| Middle East or West Asia | 26 | 1.6 |
| East Asia | 104 | 6.5 |
| Central Asia | 5 | 0.3 |
| South Asia | 84 | 5.3 |
| Southeast Asia | 55 | 3.4 |
| Polynesia or Pacific Islands | 3 | 0.2 |
| Indigenous groups | 67 | 4.2 |
| Multiple Origins | 136 | 8.5 |
| Other | 34 | 2.1 |
| Unknown Ancestry | 112 | 7.0 |

Given that our paper was interested in individuals across the lifespan, we have also provided a breakdown of ancestry across each age cohort (Table S2).

**Table S2**

*Breakdown of participant ancestry across each age cohort.*

| Ancestry | 14-17 (%) | 18-24 (%) | 25-34 (%) | 35-44 (%) | 45-54 (%) | 55-64 (%) | 65-74 (%) | 75+ (%) |
| --- | --- | --- | --- | --- | --- | --- | --- | --- |
| North Africa | 3.3 | 2.9 | 1.5 | 0.5 | 2.0 | 0.0 | 0.0 | 0.0 |
| Africa (not including North African countries) | 11.0 | 9.4 | 6.5 | 3.0 | 1.0 | 1.0 | 0.5 | 0.5 |
| Central America | 7.7 | 7.1 | 5.0 | 1.5 | 2.0 | 0.5 | 0.5 | 2.0 |
| South America | 2.2 | 2.9 | 5.5 | 1.0 | 0.5 | 0.5 | 0.5 | 1.0 |
| Eastern Europe | 14.3 | 12.3 | 10.4 | 15.1 | 17.0 | 13.6 | 17.5 | 19.9 |
| Western Europe | 12.1 | 20.4 | 22.9 | 28.1 | 39.5 | 52.8 | 53.0 | 52.2 |
| Middle East or West Asia | 4.4 | 2.3 | 1.5 | 1.5 | 1.5 | 1.5 | 1.0 | 0.5 |
| East Asia | 4.4 | 3.2 | 10.0 | 11.6 | 9.5 | 6.0 | 4.5 | 3.5 |
| Central Asia | 0.0 | 0.6 | 1.0 | 0 | 0.5 | 0.0 | 0.0 | 0.0 |
| South Asia | 9.9 | 8.7 | 9.0 | 7.0 | 2.5 | 3.0 | 0.5 | 2.0 |
| Southeast Asia | 3.3 | 5.2 | 5.0 | 6.0 | 3.0 | 1.0 | 2.0 | 1.0 |
| Polynesia or Pacific Islands | 1.1 | 0.6 | 0.0 | 0.0 | 0.0 | 0.0 | 0.0 | 0.0 |
| Indigenous groups (in North America) | 7.7 | 4.2 | 7.0 | 7.0 | 4.0 | 4.0 | 1.0 | 0.5 |
| Indigenous groups (in Oceania) | 0.0 | 0.0 | 0.0 | 0.0 | 0.0 | 0.0 | 0.0 | 0.0 |
| Multiple Origins | 13.2 | 13.9 | 10.0 | 9.0 | 6.5 | 3.0 | 5.5 | 6.5 |
| Other | 3.3 | 1.6 | 2.5 | 1.5 | 2.0 | 2.5 | 2.5 | 2.0 |
| Unknown Ancestry | 2.2 | 4.5 | 2.5 | 7.0 | 8.5 | 10.6 | 11.0 | 8.5 |

*Note.* The percentages in each column denote the proportion of a given age cohort that identifies with a certain ancestral origin. For example, 3.3% of 14-17-year-olds reported North Africa as their ancestral origin.
